# What do they say they are doing? a mixed-methods analysis of Swedish gambling operators’ duty of care action plans

**DOI:** 10.1186/s12954-025-01349-y

**Published:** 2025-12-05

**Authors:** Nathan Lakew, Philip Lindner

**Affiliations:** 1 Department of Clinical Neuroscience, Center for Psychiatry Research, Karolinska Institutet, Stockholm, Sweden; 2 Stockholm Health Care Services, Region Stockholm, Sweden

**Keywords:** Duty of care, Action plans, Responsible gambling, Rubric-based document analysis, Policy and regulations

## Abstract

**Background:**

Accountability for gambling-related harms remains a contested issue, with debates centering on who should be responsible and how interventions should be structured. While Responsible Gambling (RG) frameworks have traditionally emphasized individual responsibility through voluntary tools, growing concerns have led regulators to introduce more robust, operator-focused duty of care requirements. This study analyzes Action Plans (APs) submitted by 52 licensed Swedish gambling operators to examine how duty of care obligations are interpreted and implemented.

**Method:**

The study employed a mixed-methods approach to analyze the APs, combining qualitative and quantitative methods. The process involved a conceptual literature review, the development of a purpose-built critique instrument, rubric-based scoring, and thematic content analysis. Each AP was individually coded and scored using a five-point Likert scale (i.e., 0 – 4) across key duty of care themes. This approach enabled a comprehensive evaluation of how operators interpret, articulate, and operationalize duty of care responsibilities within the Swedish gambling regulatory framework.

**Results:**

The overall alignment scores were generally low, with a median of 1.94 and 50% of documents falling within a moderately spread range of 1.15 to 2.75; only 10% scored above 3.25. While operators performed relatively well in monitoring and risk identification (mean = 2.67), their lower scores in proactive engagement (mean = 1.62) point to a reactive, response-driven focus rather than a preventive strategy. Additionally, a prevailing narrative across the APs frames harmful gambling as affecting only a vulnerable group, thereby subtly justifying minimal and narrowly targeted interventions.

**Conclusions:**

The findings highlight the influence of RG frameworks, with many operators emphasizing individual responsibility rather than systemic harm reduction. In addition, there is a need for clearer, more robust guidelines, as higher compliance was linked to well-defined requirements. Instrumental templates, such as the critique tool developed in this study, can support designing measurable requirements and evaluating their implementation. Finally, duty of care directives can play a dual role: establishing harm reduction specifications and reshaping the narratives that define gambling. Framing them as both regulatory tools and narrative-making platforms can provide policymakers with a powerful means to reorient the discourse around gambling and enhance harm reduction outcomes.

**Supplementary Information:**

The online version contains supplementary material available at 10.1186/s12954-025-01349-y.

## Background

The question of accountability for gambling-related harms—including who should be responsible, to what extent, and how that responsibility should be defined and enforced—remains a central topic of discussion in the gambling field. Since the late 1990s [1], Responsible Gambling (RG) has provided a legal and practice-oriented framework in which secondary and tertiary interventions should ideally be offered in the form of non-intrusive, voluntary self-help tools, targeted primarily at customers with demonstrated risk. However, growing concerns about problem gambling and its social consequences have led government regulators to mandate more rigorous requirements that emphasize the operator’s responsibility to be actively involved in players’ gambling activities. These requirements, captured under the term *duty of care*, vary across jurisdictions, ranging from mandating operators to assess players’ risk to broader expectations that include proactive player engagement and preventive interventions with the goal of harm reduction [2, 3].

There exists, however, continuing debate in the field on how we problematize harms from gambling, and with it, what strategy should be developed and implemented in terms of duty of care responsibilities. As Ukhova et al. [4] noted, two key approaches have broadly shaped player protection policies: the individual-framed perspective, which focuses on personal responsibility and self-regulation; and the system-frame perspective, which emphasizes structural safeguards and regulatory interventions [5]. Traditionally associated with the so-called Reno model, the premises behind individual-framed measures problematize gambling harms as a form of subjective experience that emerges from unhealthy consumption in terms of, for example, excessive use of gambling products. Viewed through this lens, the duty of care centers on identifying risky gambling behaviors at the individual level—often by examining the relationship between gambling exposure (or ‘dose’) with signs of problematic play [6]. Intervention strategies are typically aimed at mitigating these risks by offering tools that support individuals to regulate their gambling behavior [7].

However, this framing has been criticized for overlooking the influence of gambling products themselves and the role of operators in potentially fostering addictive behavior [8]. Within the realm of the system-level approach, the public health perspective has advocated for a more stringent prevention-oriented approach, such as limiting access to gambling activities [9]. From this perspective, a duty of care would entail a focus on measures reducing the burden of gambling at the population or sub-population level in line with public health models such as the Total Consumption Theory [10). However, this perspective has also been criticized as ill-suited for the gambling context, given that the distribution of harm is highly skewed [11].

The expansion of online gambling has also drawn profit-driven third-party actors, including online entrepreneurs, game developers, and digital payment processors (e.g., fintech), at times with neither a gambling background nor safety and protection policies appropriate for this market. Since these actors are not always equally regulated under existing protection frameworks, their growing involvement has created uncharted territory for policymakers. A related challenge is the difficulty in garnering support for a broader protection agenda, especially when gambling still enjoys wide support across cultural, political, economic, and sometimes social spheres [12]. These favorable views often portray restrictive measures as limiting individuals’ freedom to choose their own recreational activities [13]. Moreover, ‘external’ factors such as financial circumstances, psychological vulnerabilities, cognitive biases, and behavioral tendenciesmay contribute to risky gambling habits [12].

Balancing all these challenges, gambling authorities across jurisdictions are expected to set a duty of care agenda and oversee its implementation. Using a purpose-built quantitative and qualitative document analysis method, the current study seeks to systematically examine gambling operators’ so-called Action Plans (APs) in which they outline their duty of care commitments when applying for a license to operate in the Swedish gambling market [14]. We were granted access to the duty of care APs submitted to the Swedish Gambling Authority (SGA) by fifty-two licensed gambling operators. Our document analysis consists of several stages, including a conceptual literature review, the development of a critique tool for evaluating and scoring the APs, and both deductive and inductive analyses of the documents. The analyses primarily examine how gambling operators have adopted and operationalized the duty of care agenda in the context of established expectations and standards. The study concludes by outlining potential future directions for the duty of care agenda.

### The Swedish gambling market

Gambling regulation in Sweden has evolved from early bans to a government monopoly and, more recently, a license-based system. Binde [15] traces Swedish gambling regulation back to the 1600 s, when it primarily aimed to prevent crime and protect vulnerable groups, including children. Gambling was banned entirely in the 1840s, then reintroduced in the early 1900s for horse racing and football games, while other games remained restricted until the late 1990s. Notably, the 1982 Gambling Act reintroduced slot machines under strict conditions and marked one of the earliest uses of RG principles in Swedish law [16].

As Alexius [17] reported, responsibility for harm reduction in Sweden gambling has also shifted over time. Initially, the state took direct responsibility for public safety by banning gambling altogether. This was followed by a phase where state-owned monopoly operators were tasked with providing gambling services while ensuring responsible practices, leading to a “re-moralization” of gambling. Growing public health concerns have expanded the role of health service providers and other stakeholders in harm prevention and support efforts. In 1989, for instance, the first Gambling Addiction Association was established, followed by a government-sponsored helpline in 1999 (*Stödlinjen*). In line with the International *Diagnostic and Statistics Manual of Mental Disorders (DSM-5)* re-classification of gambling as a behavioral addiction*,* in 2018 Sweden explicitly included gambling disorder in social and healthcare legislation.

Finally, in discussing harm reduction responsibility and duty of care in Sweden, it is important to point out the broader ‘ethos’ of the Nordic welfare system that, in general, is characterized by the delicate balancing of universalism, social resilience, and inclusion values while respect for individual autonomy [18]. Such a setting also means, as Egerer et al. [19] remarked, maintaining the status quo either through a paternalistic approach (e.g., a gambling monopoly) or a reliance on ‘good citizenship’ where both public and private entities are expected to ethically operate. This has subtly produced a system that rarely takes a strong normative stand but mostly focuses on guidelines, advice, and principles.

### The current study

In 2018, Sweden underwent a major gambling policy reform, shifting from a state-controlled monopoly to a license-based market and expanding the number of licensed operators from two to approximately 60. The SGA is the government agency responsible for ensuring the legality, safety, and reliability of the gambling market in Sweden [20]. Alongside the 2018 Gambling Act (2018:1138) and Gambling Ordinance (2018:1475), which require all licensed operators to implement measures, the SGA issued a regulatory instrument — LIFS 2018:2: Regulations and General Advice on Responsible Gambling — to clarify how duty of care responsibilities should be interpreted and implemented in practice. Together, these legal documents [21, 22] and SGA’s regulatory instrument [14] set the stage for the duty of care requirements that govern the licensed gambling market in 2019.

As part of a follow-up effort, on February 11, 2020, the SGA requested all licensed gambling operators to submit Action Plans (APs) by March 11, detailing their strategies to fulfill duty of care responsibilities accordingly with the Gambling Act 2018: Chapter 14 §1. It is worth noting that many operators licensed in Sweden in 2019 already held licenses in other jurisdictions and likely had pre-existing duty of care protocols in place. Through a freedom of information request, we obtained access to 52 of these APs. All but one of the operators held licenses for commercial online gambling and/or commercial betting, while one operator – the former monopoly provider – also held a license for land-based casino operation. This study examines how licensed gambling operators in Sweden articulated and operationalized their duty of care commitments within these regulatory submissions.

## Methods

In conducting the analysis, we used a mixed-methods approach, combining qualitative and quantitative evaluation of the documents. Specifically, we followed these steps: (1) familiarization with the documents and initial sorting, (2) developing a critique and analysis instrument, (3) applying rubric-based scoring, and (4) conducting quantitative and qualitative analysis. During these steps, each AP was taken as a unit of analysis and evaluated individually during the quantitative and thematic coding stages.

### Step one: document familiarization and simple sorting

After receiving all documents through secure cloud sharing, we anonymized the names and assigned each AP a unique identifier. Some operators submitted multiple documents, either as appendices or as separate strategy and operationalization files. These were categorized as subdocuments under the respective operator’s AP. Once the filing system was set, all the documents were converted into a PDF format and imported into NVivo 15^©^, a qualitative content analysis tool that was used to conduct qualitative coding and analysis throughout the project. The first author (NL) conducted an additional review of the documents that involved reading through the 52 APs and their corresponding attachments to gain a general picture of the material and ensure the accuracy of the content. This process allowed us to perform first-order inductive coding, which later formed the foundation for further qualitative coding.

### Step two: creating a critique and analysis instrument

Since no suitable tool exists for evaluating duty of care of APs, most of the analysis preparation focused on step two. Various analytical methods are used in the literature for document analysis, including critical discourse analysis [23], in-depth hermeneutic inquiry [24], thematic content analysis [25], and the development of customized analytical tools for examining documents [26]. It is also a common practice to prepare various data extraction sheets in advance to systematically record what is included or excluded in documents—often referred to as a critique instrument. In our analysis, we combined a critique instrument-based evaluation with qualitative thematic analysis. To develop the critique instrument, we followed the following four steps.

#### Defining the scope and objectives

We began with an inductive examination of responsible gambling guidelines, the Swedish Gambling Act, and other RG documents issued by the state during and immediately after the deregulation of the gambling environment in 2019. Specifically, we conducted a manifest content analysis of three foundational legal and regulatory documents: the Gambling Act (2018:1138), the Gambling Ordinance (2018:1475), and the Swedish Gambling Authority’s regulation and general advice on responsible gambling (LIFS 2018:2). Several recurring elements were identified in these documents, including behavioral risk assessment and monitoring procedures; the application of limits such as deposit, loss, and login time; player-facing measures such as feedback mechanisms, self-assessment tools, and self-exclusion options; internal operator practices such as documentation, escalation protocols, and customer service training; as well as requirements related to transparency in game design.

As summarized in Table 1, many of these regulatory expectations were anchored in specific legal provisions. The table outlines a selection of key elements that formed the core of the Swedish duty of care framework at the time. LIFS 2018:2 contained both binding regulations, which operators were legally required to follow, and non-binding general advice, which served as interpretive guidance to support compliance. These regulatory elements helped define the scope of our evaluation criteria and informed the keyword development strategy used in the subsequent literature search.

**Table 1:** Key Regulatory Elements of Sweden’s Duty of Care and RG Regulatory Framework (Based on 2018 Guidelines)

Legal and regulatory element	Legal reference & notes
Duty of care and RG principles Chapter 14th of the gambling act Chapter 11th of the gambling ordinance LIFS 2018:2	Gambling Act Ch. 14 §1 – 13 and Ch. 11th §1 – 12 of the gambling ordinance (binding). requires operators to monitor gambling, provide RG tools, and support players at risk
Behavioral monitoring & risk detection	Gambling Act Ch. 14 §1 (binding). continuous monitoring to detect harmful play is a core duty
Proactive engagement	Gambling Act Ch. 14 §10; LIFS 2018:2 §§10, 13, 15; gambling ordinance Ch. 11 §7 (binding). requires self-tests, real-time messages, and personalized RG prompts
Follow-up interventions	Gambling Act Ch. 14 §1; LIFS 2018:2 §§21–22 (binding). obligates escalation, referrals, and reporting of outcomes
Deposit, loss, and time limits	Gambling Act Ch. 14 §§6–7; LIFS 2018:2 §§8–9; Gambling ordinance Ch. 11 §3—5 (binding). Players must set limits; operators must enforce them
Self-exclusion	Gambling Act Ch. 14 §§11–12; LIFS 2018:2 §17; gambling ordinance Ch. 11 §8 – 12 (binding). operators must enable and promote both local and national self-exclusion
Player feedback (win/loss/play time)	LIFS 2018:2 §§12–15 (binding). requires alerts on session time, wins/losses, and exit prompts
Access to information	Gambling Act Ch. 14 §4; LIFS 2018:2 §§14–15 (binding). must display loss data, limits, and RG tools at login
Transparency in game design	Gambling Act Ch. 14 §5; LIFS 2018:2 §§19–20 (binding). prohibits deceptive design and simulative wins
Organizational responsibility	Gambling Act Ch. 14 §§13–14; LIFS 2018:2 §§4–5, 21–23 (binding). mandates staff training, internal procedures, and documentation

It should be noted that, for legal purposes, the Swedish Gambling Authority (SGA) interprets Duty of Care (i.e.,omsorgsplikt, in Swedish) to the provisions of §1, Chapter 14 of the Gambling Act—namely the identification of problem gambling and the obligation to protect players from excessive gambling by helping them reduce their play. In practice, however, the operational scope of duty of care extends beyond the narrow legal definition since the means of which the Duty of Care goal is achieved, and the definitions included, are further regulated in other paragraphs of both Chapter 14 (Responsible gambling and consumer protection) and elsewhere in the Gambling Act, e.g. limit-setting (7 §), requirement to offer self-tests (10 §), self-exclusion (11 §) and similar. For this reason – and as evident by the fact that examined action plans did not limit themselves to the exact provisions of § 1 of Chapter 14 – we utilized a wider definition of Duty of Care, more congruent with internationally prominent definitions, than the narrower legal definition.

#### Conceptual literature review

To guide the development of a scoring instrument with structured evaluation criteria, we conducted a literature review examining how regulatory elements outlined in the SGA’s guidelines, the Gambling Act, and related policy instruments have been implemented and evaluated in practice. The objective was to identify how duty of care and RG strategies are operationalized—through operator-level interventions, policy frameworks, or regulatory enforcement—in order to inform the construction of analytic dimensions. The lack of detail in this legislation and policy is a recurrent topic in Swedish gambling discourse; as currently constructed, some of the details are instead designed to be developed through court rulings. To circumvent this issue, it was necessary to conduct a broader review to identify thematic constructs, practical implementation patterns, and evaluative indicators suitable for rubric-based document analysis. The resulting dimensions were intended to reflect both the regulatory expectations embedded in Swedish policy and the practical implementation patterns found in the broader literature, enabling the development of scoring criteria at a more granular level, aligned with the complexity of the underlying regulatory aims.

In line with this ambition, we conducted a conceptual literature review, drawing on searches conducted in the following databases: Medline (Ovid), Sociological Abstracts (ProQuest), Web of Science Core Collection (Clarivate), and PsycInfo (EBSCO). The search strategy was first developed in Web of Science and then adapted for use in other databases. We structured the search using two keyword blocks: (1) Operator-Focused Block: This included combinations of terms related to gambling operators and their responsibilities including ““gambling” AND (“operator*” OR “casino*” OR “online” OR “betting”) AND (“duty of care” OR “ethical responsibilit*” OR “legal obligat*” OR “csr” OR “social responsibilit*” OR “operator responsibilit*”)) AND ((“responsible gambling” OR “player protection” OR “consumer safet*” OR “harm minimization” OR “problem gambling”) OR (“morals” OR “ethics”) OR (“industry policies” OR “strategy” OR “resources” OR “training”)) (2) Policy and Public Health Block: This focused on gambling policies, regulations, and public health laws, incorporating terms such as: Regulatory framework: “Gambling policy,” “gambling regulation*,” “gambling legislation”; Public health and consumer protection: “Public health,” “consumer protection,” “public health policy*,” “health regulat*,” “marketing*,” “consumer safet*,” “harm minimization”.

All papers published between Jan 2014 and Dec 2024 were included, and the search resulted in 1,111 peer-reviewed manuscripts. The Preferred Reporting Items for Systematic Review and Meta-analysis (PRISMA) were used to guide the search and screening procedure (See Fig. 1 in Additional file 1). We imported all references into the Covidence^©^ – a collaborative literature review application – for screening and started with a review of abstracts, and 136 articles were selected for full-text review.

Studies were included if they (1) examined how RG strategies or duty of care requirements are operationalized in practice—through operator-level interventions, system-level policies, or regulatory implementation, (2) provided clear, actionable indicators or frameworks related to monitoring, engagement, or harm reduction, (3) offered empirical findings, policy analyses, or structured reviews of RG tools/strategies, and (4) were published in peer-reviewed journals. Studies were excluded if they (1) lacked applied or strategic content (e.g., philosophical commentary or general opinion pieces), (2) focused solely on theoretical discussions or conceptual framings of responsible gambling, or (3) did not address implementation mechanisms or methods of operationalizing duty of care.

After abstract screening (n = 994) and a full-text review (n = 136), 59 studies met the inclusion criteria and were selected for synthesis. All screening and data extraction review steps were performed by the lead author (NL). Given the scope of the review and the relatively homogenous nature of the literature, this approach was considered to carry a low risk of bias while supporting conceptual consistency during thematic coding. This was followed by an inductive content analysis of the included studies (see Table 2 in Additional File 1, p. 3 for full bibliography). Articles were initially categorized by subject focus (e.g., login session limits, bet limits, withdrawal limits) and later grouped under broader thematic categories (e.g., limit-setting and control). This iterative process informed the development of a structured extraction sheet (see Table 3 in Additional File 1, p. 6), which was reviewed by a co-author before final extraction. Full data extraction was subsequently carried out by the lead author, with attention to thematic indicators, implementation mechanisms, and evaluation criteria relevant to duty of care and/or RG strategies. This process resulted in the identification of eight core duty of care themes, including their key constructs, indicators, and implementation strategies (see Table 2 for a summary of the themes). The full conceptual literature review results, including detailed constructs, indicators, and sub-themes for each dimension, are provided in the Additional File 1, Table 5, pg 7.

**Table 2:** Summary of duty of care dimensions identified through conceptual review

Rubric dimension	Description	Examples of implementation
Risk identification and Monitoring	Detecting problematic play through behavior tracking	Session tracking, stake analytics, predictive models, and periodic risk surveys
Player protection and safety procedures	Structural safeguards to minimize harm	Self-exclusion, credit bans, bonus restrictions, and withdrawal limits
Limit setting and control	Tools for player-imposed limits on activity	Deposit caps, cooling-off periods, budget calculators, and voluntary pre-commitment
Proactive engagement	Personalized outreach to promote responsible gambling	Alerts, RG reminders, pop-ups, self-assessment prompts, and age-sensitive messaging
Access to information and awareness	Easy access to gambling behavior data and RG info	Loss/win summaries, account history, RG tool access
Follow-up and escalation	Tiered intervention based on severity	Phone/email contact, feedback loops, referrals, and prevention center collaboration
Product strategy and transparency	Clear risk info and healthy product guidance	Game risk disclosures, RG labels, product design protocols
Training and customer service readiness	Staff capacity to respond to RG risks	Mandatory RG courses, escalation protocols, and behavior recognition training

#### Designing the critique instrument and establishing a database

Different scoring methods can be used to systematically evaluate policy and document analysis. In this study, we applied a rubric framework to create a structured scoring system with defined, measurable criteria and a scaled rating approach. Rubrics allow flexibility—such as developing tailored questions for each theme—while ensuring a consistent and transparent scoring scale that supports interpretive clarity and comparability across cases [27]. We applied a rubric scaling system to establish the critique instruments as follows.

We began by developing a set of content analysis questions for each theme based on results from the literature review. The data extraction sheet of each of the articles provided rich content to develop such a set of questions. For example, under the *Risk Identification and Monitoring*, initial sub-questions included, but were not limited to: ‘Does the AP describe how risky behaviors are defined and identified?’, ‘Are there mechanisms in place to mitigate harm once risk is detected?’, or ‘Does it specify ongoing monitoring processes for gambling behavior’? Next, these sub-questions were iteratively refined and synthesized into a single primary scoring question for the dimension. This process was repeated for all themes until each theme was assigned a scoring question to measure its objectives using an appropriate indicator(s) (e.g., effectiveness, comprehensiveness, relevance, tenability). This allowed for greater conceptual precision as well as flexibility than using a generic scoring prompt across all dimensions.

Third, we adopted a Likert-style scale to provide room for subjective judgment while maintaining systematic comparability. During piloting the scaling, we found that some APs entirely omitted certain themes. As a result, the initial 4-point scale (1 = Poor to 4 = Excellent) was revised to include an additional category for absence (0 = Missing). The final scoring codebook thus featured a 5-point Likert scale (0 = Missing, 1 = Poor, 2 = Fair, 3 = Good, 4 = Excellent) – (See Table 6 in Additional file 1, p. 10).

### Step three: scoring of the documents

We used the Nivio 15 application to go through the APs while using the tailored instrument to score the action plans systematically in a separate Excel sheet. We started by registering the ‘General physical attributes of the documents’ into the Excel sheet manually, including the number of pages, appendices, figures, and tables. We then evaluated each AP against scoring questions developed for the eight rubric themes. Scores were assigned on a five-point scale (0–4), ranging from “Missing” to “Excellent,” based on how well the AP addressed the theme’s objectives. For example, under ‘Risk Identification and Monitoring,’ scores reflected whether the AP outlined a coherent, data-driven approach using behavioral analytics, predictive modeling, and early intervention protocols.

The first author (NL) carried out the initial scoring and qualitative coding. To assess scoring reliability, the second author (PL) was randomly assigned (through a random seed supplied by a researcher not otherwise involved in the study) five APs to score independently using the same scoring codebook (three APs were dropped from consideration due to conflicts of interest, see below). Due to the five-step nature of the rating scale, Cohen’s weighted κ was chosen (with quadratic weights) as a measure of inter-rater reliability. Run on all 8 × 5 ratings, a weighted κ of 0.80 was achieved (95% CI: 0.67–0.93), revealing substantial agreement. Moreover, the average inter-rater difference score across all items and APs was M_diff_ = 0.05, with the 95% confidence interval including zero (-0.23–0.3), suggesting no scoring bias in any direction.

### Step four: conducting quantitative and qualitative analysis

The final quantitative scores (See Table 7 in Additional file 1, pg.14) were then exported to SPSS (version 28) and the R (4.4.1) statistical environment for data analysis and visualization. The quantitative analysis focused on identifying patterns, trends, and variations across different rubric dimensions. In addition, summaries around how each dimension was scored within and across the operators, and correlation analyses were performed to assess relationships between different scoring dimensions and identify potential factors influencing duty of care implementation. The qualitative content analysis followed a two-step approach: manifest and latent content analysis. In the first step, manifest content analysis began with fully inductive coding (Step 1) and later continued alongside the quantitative evaluation of the documents (Steps 3 and 4) to further enrich the categories identified through quantitative analysis. Finally, we concluded our analysis with latent content analysis, interpreting and extrapolating the underlying meanings within the formulation and content of the APs. This approach allowed us to infer operators’ assumptions and analyze their strategic focus through the lens of existing literature. The current study will use insights from the latent content analysis in the discussion and implications sections.

## Results

### Statistics and quantitative findings

In total, more than 929 pages of documents were examined, including appendices. The average length of the AP documents was 18 pages, varying from half a page to 47 pages. Of the 52, 14 APs were submitted with an additional appendix with content varying from duty of care email templates and examples of real-life message exchanges to documentation of the operator’s overall RG strategies, proprietary RG system dashboard, marketing policy, and payouts, as well as anti-laundry procedures. Although length does not fully capture the quality across rubric dimensions, we found a strong correlation (r = 0.71, p < 0.001) between the number of pages and the score, indicating a tendency for longer documents to receive higher scores.

The mean scores across the eight regulatory dimensions revealed a stronger emphasis on monitoring and risk identification (2.67) and player protection (2.65), as shown in the histograms (See Figure A1). However, significant variability exists across the categories, ranging from 0.44 (Product offering selection strategy and transparency dimension) to 2.67, reflecting inconsistencies in regulatory implementation. Lower scores in areas like proactive engagement, access to information, and product transparency suggest a prioritization of risk assessment over proactive engagement and transparency practices (See Figure A2). Internal consistency was calculated to α = 0.92 (95% CI: 0.88–0.95); with the exception of the *Product offering selection strategy and transparency* item, inter-item correlations were strong (see Figure A3). This indicates a single underlying component corresponding to a duty of care alignment and also validates the use of the total score. Results of a parallel analysis to examine component structure supported this interpretation, with a single component accounting for 66% of the variance.


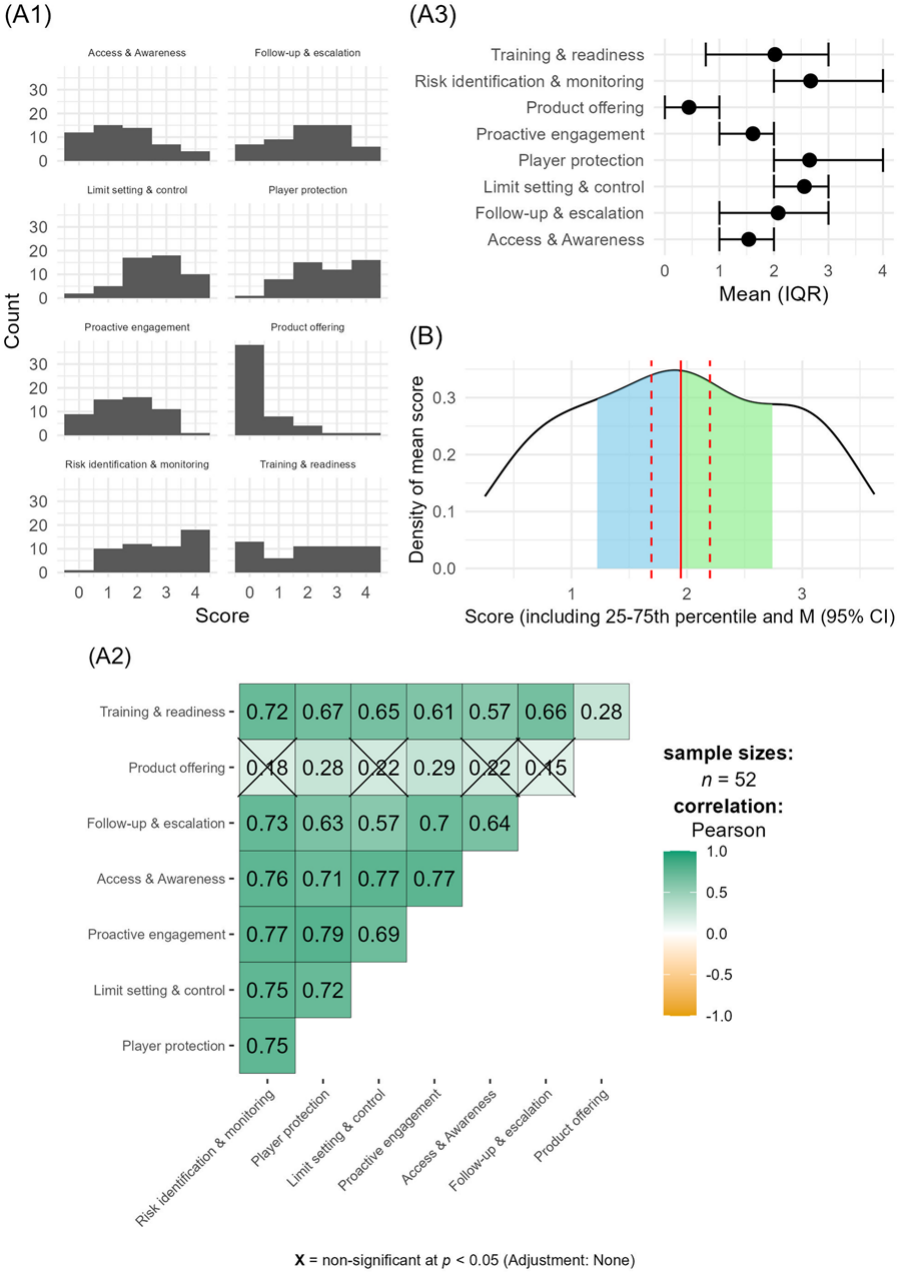


*(A1) Histograms of each item’s score. (A2) Means and IQR of different items/dimensions. (A3) Inter-item correlation heatmap. (B) Density plot of average score, including mean and 95% bootstrapped (k* = *10,000) 95% confidence intervals, as well as IQR marked in blue and green (second and third quartile, respectively).*

The interquartile range of the average score (1.15–2.75, out of a possible interval of 0–4) revealed that the middle 50% of scores were moderately spread: one-quarter (25%) of the scores were at or below 1.16, meaning one-quarter of the submitted document consists of relatively low-scoring APs. While only 10% of documents scored above 3.25 (out of a possible 4), a similar percentage scored below 0.62. The median score of 1.93 further supports the conclusion of a generally low level of alignment with duty of care expectations. A density plot of the total scores, including the mean (with 95% CI) and interquartile range, is shown in Figure B.

### Risk identification and monitoring dimension

Most operators devoted extensive sections of their APs to outlining their gambling risk assessment strategies (18 of 52; 34.6% scored 4). Fifteen operators claimed and presented an in-house proprietary system they use to track players’ gambling behavior, while others highlighted the use of data analytics tools such as Amplitude, Jira, and Tableau to create a summary of spending and withdrawal patterns for detecting risky behavior. In addition, integration of RG efforts with customer service (CS) tools such as Zendesk was noted, and two APs reported partnerships with third-party data analytics providers for behavioral tracking and risk identification. Most operators applied a three to four-level risk scoring method to evaluate their players: No risk, moderate risk, high risk, and problem gambler (the last two sometimes merged into one). The common method of identifying risk focused on profiling individuals based on behavior data, even though some noted communication with the players and, at times, third-party referral (e.g., family members) can lead to risk classification. However, we also found that some APs, particularly those with medium to low scores in this dimension, showed inconsistencies in defining risk, often relying on normative statements rather than outlining concrete strategies. Finally, seventeen operators identified suicide as a significant risk indicator, with eleven of them also referencing self-harm.

The discussion on risk identification and monitoring across the APs primarily focuses on matching customer behavior with recognized markers of problem gambling. Risk is typically assessed through product usage patterns (e.g., time spent, frequency, intensity), payment markers (e.g., deposits, bets, withdrawals, hitting limits), and customer service interactions (e.g., limit change requests, lack of correspondence). It is important to recognize, however, that this approach largely reflects existing regulatory and research frameworks that prioritize behavioral indicators for risk detection. While such alignment is understandable, scholars have also noted that these framings may be selectively reinforced by industry actors [28, 29]. Overall, these dynamics highlight the need for more comprehensive regulatory and research guidance that addresses both individual behavior and product design as drivers of harm.

### Players’ protection and safety procedures dimension

As the second-most covered dimension (16 of 52; 30% scored 4), *player protection and safety* commonly included measures such as age verification, moderation in marketing, exclusion (including operator-imposed bans), gambling activity updates in the form of popups and alerts (featuring e.g., time spent), and RG help pages. Implementation varied, however, with more elaborate and interactive measures found among operators with higher scores, while lower-scoring operators tend to adopt static approaches (e.g., an informational RG page) with less frequent interaction—primarily to meet explicit compliance requirements. In the former case, we found evidence of multi-layered safety protocols, including personalized risk alerts and real-time pop-ups. In the latter group of operators, however, we observe a tendency to place the burden of being responsible on the players (e.g., paraphrased quote for anonymity: *‘At (company name), we recommend you use gamban or netnanny to protect underage gambling and for a safety gambling’* – an AP scored 2).

### Limit setting and control dimension

This dimension involves providing and enforcing limit-setting tools, including financial caps (deposit and withdrawal limits), safeguard measures (game-specific time and session restrictions), and occasionally additional resources like budget calculators, while also contacting customers when specific thresholds, such as deposits exceeding 10,000 SEK per month, are surpassed. Compared to other dimensions, limit setting achieved a mid-range performance, with most scores falling between 2 and 3, indicating that operators generally met basic requirements but rarely exceeded them. However, there were notable discrepancies in how operators implement and enforce these measures. Higher-scoring operators (3–4) typically described a wider range of limit-setting tools, including financial, session-based, single limit setting across the brands, and default limits based on player data, as well as pop-up alerts and cooldown periods. In contrast, lower-scoring operators focused primarily on basic deposit caps, expecting customers to contact them regarding limit setting and customer outreach only when those limits are exceeded.

Across operators, we observed that ‘enforcing’ limit commitment typically applies when the operator is unable to reach the customer. If a customer confirms they can manage a limit increase or justify a large amount of spending as within their means, they are often allowed to bypass the limits. This is concerning, as those experiencing gambling problems may be less likely to withhold consent. For instance, this paraphrase excerpt shows how low a threshold can be to change a pre-commitment limit setting: ‘*These deposit limits may be changed at any time, and may also be made less strict, given customer consent (AP scored 2)... If the player stated that they are in control of their gambling and can afford it, they are allowed to increase the limit’ (an AP scored 3).*

### Proactive engagement dimension

Proactive engagement provides a platform for operators to step out of the requirement thinking and encourage players with tools that promote healthy gambling practices. Such initiatives can include interactive educational programs, gambling activity check-ins, personalized reminders, and targeted messages. Most operators scored under 2 in this dimension (67%; with 9 scoring 0 and 1 scoring 4). Such a result is consistent with operators’ focus on sticking to fulfilling explicit requirements, as noted in the following paraphrased excerpt: ‘*If a player raises a limit or sets a higher limit for deposits than 10.000 SEK per month, we will contact the play *via* email to make sure the player is playing within their means of income in order to fulfill our duty of care requirements* (an AP scored 1). Unique to higher-scoring APs, we observed proactive engagements such as ‘exclusion-returnees’ programs, targeted outreach strategies, tailored interventions, and interactive educational initiatives (e.g., demo games). Some also describe implementing proactive affordability checks in collaboration with external financial institutions and payment agencies.

### Access to information and Awareness dimension

In line with the proactive engagement dimension, the *access to information* dimension reflects attempts to provide players with actionable insights into their gambling behavior to encourage responsible gambling. To achieve this, operators must have the appropriate infrastructure to store gambling histories, effective communication channels to share this information with players, and systems in place to analyze player behavior using these datasets. This dimension showed low alignment, with 78% of operators scoring below 2, including 12 who scored 0. Most operators lacked a clear strategy for storing gambling history and sharing information with players, often presenting it in ways that are difficult to access or understand. In contrast, higher-scoring operators tend to regularly update players on their gambling activity and ensure that gambling history can be easily accessible from the account profile and integrated into the user experience. These operators also tend to share behavioral data with the academic community. We also observed that this group includes structured reporting of account data and funding support for broader RG initiatives as part of their practices.

### Follow-up Interventions and escalation of problem gambling severity levels dimension

This rubric's dimension, strongly correlated with the risk assessment and monitoring theme (r = 0.728), focused on implementing a structured plan to follow up with players who are deemed at risk and/or are currently receiving interventions. The overall APs scoring shows that most operators received 2–3 (28% each; with 6 and 7 APs scoring 4 and 0, respectively). Operators in the higher end (3 or 4) tend to implement a structured escalation pathway (tiered), where at-risk players receive progressively stronger interventions based on their behavior.

Escalation procedure varies across the operators: escalating players between different teams (customer service, RG, Legal) with a tag (often green, yellow, and red), RG team escalating players from limiting deposit to exclusion, and at times from providing RG tool up to referral and partial payment for therapy programs. Model-based escalation was found in all APs scored 4, as noted in the following paraphrased AP_32 document: ‘*We applied eight escalations following up measures starting with provision of RG information and applying limits to reality check tool, break and timeout, CS intervention, self-exclusion, and ban’*. Lower-scored APs tended to describe limited or general measures, at times lacking clear escalation procedures or relying primarily on automated messaging systems with little indication of direct customer service (CS) involvement.

### Product offering selection strategy and transparency dimension

Most operators had the lowest score in the *product transparency* dimension (73% scoring 0, while only 2 APs scored 3 and 4), which, among other things, focused on clearly communicating the risks associated with game features, erroneous beliefs in the chance of winning, and providing educational resources regarding game products. The most frequently cited resources under this theme included RG information pages, brief game-related content, and general policy statements (e.g., Paraphrased: ‘*We ensure that the games are designed in compliance with laws and regulations’).* Two of the APs with a higher score have a structured method of selecting games that includes a checklist for rating, follow-up on risk parameters, and demo games for educational purposes (e.g., Paraphrased: ‘*we provide demo games with educational purposes with the same random outcome as the real stakes’).*

### Training and customer service readiness dimension

We found that most operators (75%) describe efforts to develop *the training and CS readiness* theme, with an average mean score of 2.02, though the theme was entirely absent in nearly 25% of the action plans despite being explicitly mentioned under the RG heading of the Swedish Gambling Act. Higher-scoring operators described comprehensive training plans, including detailed training modules, certification processes, and the use of dedicated intranet pages for ongoing guidance. These operators also emphasized regular training (e.g., Paraphrased: ‘*Staff are tested annually, requiring an 80% pass rate*’) and extended training across multiple departments (e.g., Paraphrased: ‘*training will be provided to board managers, RG staff, CS, sales, and to some level all staff*’). Several operators reported collaborating with third-party providers to deliver training sessions.

Additionally, training manuals often emphasized risk assessment and outlined the corresponding actions to be taken—covering areas including player risk classification, escalation protocols, contact procedures, and in some cases, templates for communication. Finally, lower-scoring operators typically offer basic and infrequent training and tend to focus on providing short onboarding sessions for new hires. In some cases, the descriptions provided were broad or abstract, making it difficult to assess the practical implementation of competence-building efforts (e.g., Paraphrased: ‘*Training according to Chapter 14, § 14 of the Gambling Act (2018:1138) must at least include the following elements*... (operator name) *shall keep a list of people who have completed the training’*).

## Discussion

Our primary findings indicate that most operators demonstrated limited alignment with duty of care requirements, particularly in areas requiring proactive engagement with players. Consistent with historical emphasis on individual responsibility, operators generally performed better in dimensions centered on risk assessment and player protection. This pattern also reflects the narrower legal definition of duty of care under Swedish law, which in practice was operationalized mainly through self-managed RG tools such as limit-setting, self-exclusion options, pop-ups, information pages, and staff training to support these measures. Some operators interpreted their duty of care as being limited to monitoring and providing self-management tools, thereby transferring the responsibility for their use to the player. Word frequency analysis identified ‘responsibly’ as the most frequently occurring word, appearing more than 2,600 times, followed by ‘information,’ which appeared over 900 times in various forms, including ‘informative,’ ‘providing information,’ and ‘informational.’

As Heidegger [30] noted, specific ways of questioning a problem can ‘build the way’, influencing how we interpret a given phenomenon and determining what constitutes an appropriate response. In reviewing the APs, we observed a dominant individual-oriented narrative in how operators problematize gambling, reinforcing a mode of questioning that selectively legitimizes focus on specific dimensions of duty of care and a group of individuals. This problematization often begins with the premise that ‘only a few have gambling problems,’ with these labeled as ‘vulnerable’ (120 word-count), ‘problematic’ (105 word-count), or described using phrases like ‘those who lose control’. Such framing not only characterizes the issue in terms of individual self-control but also confines it to a narrowly defined minority. Once the problem is framed as affecting only a few, the corresponding response is positioned as necessarily limited, non-intrusive, and designed not to disturb the broader population of ‘healthy’ players. This is despite evidence indicating a strong correlation between operators’ revenue and the extent of individuals classified as problem gamblers [31].

Additionally, by “signposting” specific groups [32], such narratives can justify a limited scope of interventions and reinforce the perception of gambling as inherently safe. By framing personal choice—rather than the product itself—as the central problem and solution space, the omission of gambling products as a potential source of harm can appear acceptable. This was evident from the limited attention given to the product offering selection strategy and transparency dimension, which received the lowest alignment rating (mean score of 0.44), in contrast to the risk assessment dimension (mean score of 2.67). These findings are also consistent with the broader scarcity of research on the role of behavioral-laden design techniques, revenue models, and gambling product characteristics [33]. As such, additional research employing framing theories and examining gambling narratives is needed to broaden the duty of care discussion beyond its current focus on individual responsibility [29, 34]. Such research should also emphasize operators’ responsibilities for prevention and harm reduction related to their products.

Our analyses also reveal that some public health-oriented measures—such as age verification, player identification, bans on underage marketing and credit gambling, etc.—were explicitly mentioned by nearly all operators, reflecting these measures’ mandatory and explicit status within the general responsible gambling directives. This suggests that operators tend to comply with stricter requirements when these are supported by clearly mandated policies and enforcement mechanisms, rather than relying solely on voluntary adherence. As Abbott [35] noted, harm reduction measures reflecting a public health-oriented approach require strong policy backing, possibly supported by independent monitoring and enforceable remedies for breaches. Other measures could also include policy-backed data sharing between researchers and operators to enhance player protection and operator accountability [36]. In addition, as Flore-Pajot et al. [37] pointed out, gambling operators should adopt a more proactive approach by not just offering RG tools but also actively encouraging their effective use among players.

Finally, we have observed operators employing various legitimacy-seeking strategies to portray themselves as compliant with regulatory requirements, especially regarding dimensions that require proactive operator engagement. Two common approaches identified were referencing prominent researchers to substantiate their action plans, as well as making future-oriented commitments aligned with duty of care expectations. Several operators devoted considerable pages of the APs to outlining future plans using phrases such as ‘going forward’, ‘future improvements we plan’, a project is underway to develop’, and ‘planned introduction of a new system for Swedish players’. At times, some APs read more like summaries of responsible gambling literature than operational documents, containing broad normative statements and general commitments rather than concrete, actionable strategies. Such evidence aligns with longstanding discussions regarding gambling operators’ tendency to engage ‘symbolically rather than substantively’ with RG initiatives that mostly aim to manage perceptions of legitimacy [28]. In addition, these findings emphasize the need for careful evaluation of action plans and independent oversight, for instance, through collaborations with the academic research community.

### Implications and future research

A key implication of these findings is that duty of care and responsible gambling directives shape how gambling harm and protection measures are framed, creating both challenges and opportunities for policymakers. On the one hand, regulators like the SGA must balance consumer protection with oversight of a legal gambling market – in contexts like Sweden – where the state owns/has a stake in major gambling operators and gambling generates significant public revenue (SEK 6.13 billion in 2024). As public health measures curb gambling activity, they may also reduce state income, creating tensions between harm reduction and revenue generation (8).

On the other hand, duty of care directives can serve as important levers to reframe responsible gambling practices. The current analysis suggests several potential pathways for doing so. For example, integrating pre-screening affordability checks as a proactive, standardized component of the player protection and safeguards dimension could enhance early intervention. In our analysis, 15 operators referenced affordability testing—typically as a reactive measure triggered after signs of risky behavior. Making this process pre-emptive could strengthen player protection, although privacy and financial data regulations currently limit direct implementation [38]. Broader coordination with authorities such as the Ministry of Finance may therefore be required to operationalize this measure within future updates of the Gambling Act.

The *Follow-Up interventions* dimension could likewise be strengthened by requiring operators to facilitate, rather than simply suggest, referral to appropriate support services. Evidence indicates that health-service providers are primarily responsible for prevention activities [39]; involving operators more actively in this process could extend the reach of early intervention efforts. Similarly, the access to information dimension might be enhanced through policy-based data-sharing arrangements between operators, researchers, and regulators. Some jurisdictions, such as Switzerland, have introduced similar requirements to improve transparency and evidence-based oversight [36].

In addition, there may be merit in further standardizing certain duty of care requirements to promote consistent interpretation and application across the sector. Our analysis identified variations in how operators addressed similar regulatory expectations. At present, such inconsistencies are often resolved through court rulings—a lengthy process that can delay harm-reduction outcomes. Introducing clearer, template-based criteria for each duty of care theme could provide a more efficient compliance pathway. The rubric developed in this study may serve as a conceptual foundation for such efforts. However, further work is needed to establish comprehensive, criteria-based guidelines that provide clearer and more consistent directions for operators. Such an approach could incorporate explicit performance indicators, routine monitoring, and defined consequences for non-compliance—measures that not only strengthen accountability but also address patterns of legitimacy-seeking behavior, aligning duty of care more closely with a public health–oriented framework.

The research field, likewise, needs to strengthen its efforts in offering practical, operationally focused guidance for policymakers. While public-health approaches to gambling harm have expanded, empirical evidence on how to embed these within operators’ compliance systems remains limited. Strengthening collaborations between regulators, researchers, financial institutions, community organizations, and healthcare services [12, 40–42] could enhance both accountability and innovation in harm-reduction practices.

Finally, future studies could examine which provider-level factors are associated with stronger implementation of player-protection measures. In our dataset, higher scores were often linked to larger, globally operating companies with broader product portfolios and more formalized staff-training structures. Comparative research across jurisdictions could help clarify how organizational capacity and regulatory context interact to shape effective duty-of-care practices.

### Limitations of the current study

This study has several limitations. First, the evaluation relied exclusively on written duty of care reports submitted by operators, rather than on observed practices. As a result, the ratings may not fully capture how these commitments are enacted in day-to-day interactions with players. Given the legitimacy-seeking behavior discussed earlier, our assessment is that operators’ real-world actions may align with or fall short of the commitments outlined in their plans. Further research is needed to address this limitation, including follow-up studies on both duty of care action plans and their practical implementation.

Second, while duty of care requirements were legally enforceable under the Gambling Act, the specific format and structure of the action plans allowed for substantial flexibility during the early implementation phase, when the regulatory framework was still evolving. Consequently, the rating results may not fully reflect the current maturity of the Swedish gambling market. However, licenses were granted for a maximum of five years, and earlier versions of the duty of care guidelines and the 2018 Gambling Act still align with the core principles of today’s requirements; legal developments since then have focused on gaps and details. As a result, the narrative of individually focused measures continues to shape the approach to duty of care and responsible gambling practices.

A further limitation concerns the construction and application of the scoring instrument. The eight dimensions in the rubric were developed based on a conceptual literature review that used the regulatory framework to define the review scope. To support conceptual clarity, each relevant unit of text in the operator reports was assigned to a single primary dimension, based on its dominant function or regulatory intent. While this approach improved consistency, we acknowledge that certain elements — such as early detection strategies or behavioral flags — could plausibly fall under more than one dimension (e.g., monitoring, engagement, or follow-up). This overlap is particularly evident when operators use a single resource to address multiple regulatory expectations. For example, one company implemented demo games both to increase product transparency and as a proactive engagement tool for individuals returning from self-exclusion — reflecting the inherently cross-cutting nature of some features. To mitigate this challenge, the rubric was designed with tailored scoring questions for each dimension, rather than applying a generic assessment question across all themes. These dimension-specific prompts were aligned with the distinct regulatory and conceptual intent of each category, which helped reduce ambiguity and improve internal validity. Nevertheless, some degree of overlap between dimensions is likely unavoidable due to the integrated nature of RG practices. We acknowledge this limitation, as conceptual boundaries between categories may be interpreted differently by other researchers. In this study, we prioritized coding consistency and mutual exclusivity to reduce bias in scoring, rather than attempting to capture all possible overlaps. Future refinements could include reclassifying dimensions or validating the framework in other regulatory contexts to enhance interpretive precision. We welcome continued dialogue and critique to strengthen the tool’s practical and analytical utility.

## Conclusions

As societal awareness of the potential harms associated with gambling increases, governments are introducing stronger measures in the form of duty of care requirements, placing greater responsibility on operators to engage proactively with players and promote safer gambling practices. Using a mixed-methods document analysis, this study examined the duty of care action plans submitted by licensed gambling operators in Sweden. The primary objective was to assess how operators interpret and operationalize the duty of care agenda, and the extent to which their plans align with established regulatory expectations.

The results show that while most operators formally acknowledge duty of care obligations, their responses remain primarily focused on RG measures, emphasizing individual responsibility. This orientation was reflected in the prominence of self-management tools such as limit-setting, self-exclusion, and self-assessment, which place the burden of behavioral change largely on the player. Framing problem gambling as an issue confined to a small, vulnerable minority appeared to legitimize narrowly targeted and non-intrusive interventions, diverting attention from structural or product-level determinants of harm. Compliance tended to be stronger in areas with clear and enforceable regulatory guidance (e.g., underage gambling), whereas other commitments appeared more symbolic than substantive. In addition, high inter-item correlations suggest that duty of care reflects a single, underlying factor: operators who scored high on one dimension tended to score high on others, and vice versa, reflecting different levels of ambition among operators.

These findings underscore the extent to which the effectiveness of duty of care as a harm-reduction mechanism depends on the clarity, enforceability, and evaluability of it's regulatory requirements. As Van Schalkwyk et al. [29] note, framing plays a crucial role in shaping the broader policy discourse around gambling—particularly in how responsibility for harm and the role of operators are defined. As such, duty of care should be understood not only as a compliance framework but also as a tool for influencing how gambling-related harm is conceptualized and addressed. In this sense, regulators designing duty of care frameworks should be mindful that, beyond setting enforceable standards, they also shape how gambling-related harm and responsibility are framed in their respective jurisdictions.

Finally, ensuring that duty of care requirements are formulated in measurable, outcome-oriented terms would facilitate systematic evaluation and continuous improvement. This study contributes to that goal by proposing a rubric-based analytical framework that can be further refined and validated across jurisdictions. Future research could build on this approach to explore how variations in regulatory design and institutional context influence the practical implementation and effectiveness of duty of care policies.

## Supplementary Information

Below is the link to the electronic supplementary material.

Supplementary Material 2

## Data Availability

Statistical code is available from the corresponding author. The analyzed documents were made available to the researchers through a freedom of information request to the Swedish Gambling Authority under the condition of preserved inter-governmental confidentiality. Requests for material should be directed to the Swedish Gambling Authority: [registrator@spelinspektionen.se](mailto:registrator@spelinspektionen.se).

## Abbreviations

AC: Action plan
RG: Responsible gambling
SGA: Swedish gambling authority
