# Supplementary material for: What do they say they are doing? a mixed-methods analysis of Swedish gambling operators’ duty of care action plans
Source: Harm Reduct J. 2025 Dec 5;22:196. doi: 10.1186/s12954-025-01349-y (PMC12679775; doi:10.1186/s12954-025-01349-y)
Supplement: Supplementary file 1 — Supplementary Material 2 [file 12954_2025_1349_MOESM2_ESM.pdf]

Supplementary Appendix

Table of content*[1]

Figure 1: PRISMA - Duty of care critique instrument **2**

Table 2: Conceptual Literature review Bibliography (included) **3**

Table 3: Data extraction Sheet **6**

Table 5: Conceptual literature review result – Duty of care themes **7**

Table 6. Scoring code book **10**

Table 7. Rubrics Dimension Results **14**

[1] Noted that Table 1 and Table 4 are included in the body of the manuscript

Figure 1. PRISMA - Duty of care critique instrument

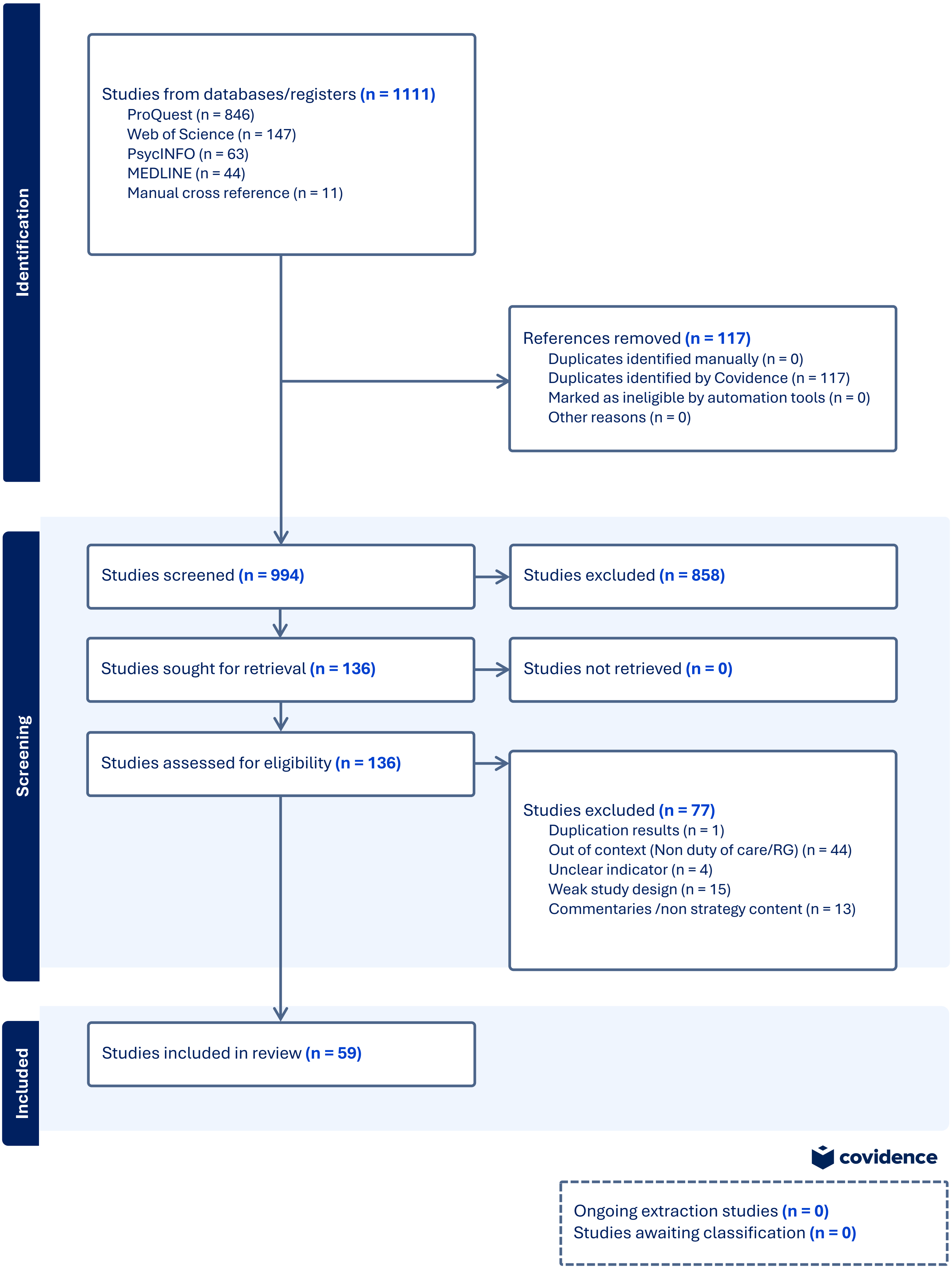

Table 2. Conceptual Literature review Bibliography

Title	Authors	Published Year	Journal	Covidence #
**Three Contrasting Accounts of Electronic Gambling Machine Related Harm: Impacts on Community Views Towards Gambling Policy and Responsibility.**	Myles, Dan; O'Brien, Kerry; Yucel, Murat; Carter, Adrian	2024	Journal of gambling studies	#1000
**A Critical Review of the Harm-Minimisation Tools Available for Electronic Gambling**	Harris, A; Griffiths, MD	2017	Journal of gambling studies	#1062
**What Behaviours and Cognitions Support Responsible Consumption of Gambling? Results from an Expert Survey**	Hing, N; Russell, AMT; Hronis, A	2017	International journal of mental health and addiction	#1063
**Responsible gambling: a synthesis of the empirical evidence**	Ladouceur, R; Shaffer, P; Blaszczynski, A; Shaffer, HJ	2017	Addiction research & theory	#1068
**Self-directed interventions for gambling disorder**	Abbott, MW	2019	Current opinion in psychiatry	#1070
**Behavioural Tracking and Profiling Studies Involving Objective Data Derived from Online Operators: A Review of the Evidence**	Delfabbro, P; Parke, J; Catania, M	2024	Journal of gambling studies	#1084
**Whose Responsibility Is It to Prevent or Reduce Gambling Harm? A Mapping Review of Current Empirical Research**	Akçayir, M; Nicoll, F; Baxter, DG; Palmer, ZS	2022	International journal of mental health and addiction	#1085
**Warning messages for electronic gambling machines: evidence for regulatory policies**	Ginley, MK; Whelan, JP; Pfund, RA; Peter, SC; Meyers, AW	2017	Addiction research & theory	#1086
**Harm reduction in gambling: a systematic review of industry strategies**	Tanner, J; Drawson, AS; Mushquash, CJ; Mushquash, AR; Mazmanian, D	2017	Addiction research & theory	#1094
**Towards a Systems-Based Responsible Gambling Approach to Mitigating Harmful Gambling: Delineating Industry's Role in Gambling Safety**	LaPlante, Debi A; Nelson, Sarah E	2023	The Journal of Gambling Business and Economics	#1101
**Clarifying responsible gambling and its concept of responsibility**	Blaszczynski, Alexander; Shaffer, Howard J; Ladouceur, Robert; Collins, Peter	2022	International Journal of Mental Health and Addiction	#1103
**The impact of personalized feedback interventions by a gambling operator on subsequent gambling expenditure in a sample of Dutch online gamblers**	Auer, Michael; Griffiths, Mark D	2023	Journal of Gambling Studies	#1104
**Effects of prevention and harm reduction interventions on gambling behaviours and gambling related harm: An umbrella review**	McMahon, Naoimh; Thomson, Katie; Kaner, Eileen; Bambra, Clare	2019	Addictive Behaviors	#1105
**Building a Responsible Ecosystem: Examining Trust and Responsibility in the Gambling Industry: Bournemouth**	Bolat, E; Arden-Close, E; Ali, R	2019	Bournemouth University Publishment	#1107
**Responsible gambling: a scoping review**	Reynolds, Jennifer; Kairouz, Sylvia; Ilacqua, Samantha; French, Martin	2020	Critical Gambling Studies	#1108
**'Of course we make money, but it has to be in a responsible way': Safer gambling practices of state-owned gambling operators**	Newall, Philip; Whybrow, Allegra Katharine; Torrance, Jamie		OSF preprint	#1109
**Safer by design: Building a collaborative, integrated and evidence-based framework to inform the regulation and mitigation of gambling product risk.**	Delfabbro, Paul; Parke, Jonathan; Dragecvic, Simo; Percy, Chris; Bayliss, Richard	2021	Journal of Gambling Issues	#1110
**Interventions to reduce the public health burden of gambling-related harms: a mapping review. Lancet Pub Health 6: e50–e63**	Blank, L; Baxter, S; Woods, H; Goyder, E	2021	Lancet Public Health	#1111

**Voluntary Self-Exclusion and Contingency Management for the Treatment of Problematic and Harmful Gambling in the UK: An Exploratory Study**	Zolkwer, Morgan B; Dymond, Simon; Singer, Bryan F	2023	Healthcare	#149
**Gambling harm prevention and harm reduction in online environments: a call for action**	Marionneau, Virve; Ruohio, Heidi; Karlsson, Nina	2023	Harm Reduction Journal	#161
**'We are not the ones to blame'. Gamblers' and providers' appraisal of self-exclusion in Germany**	Kraus, Ludwig; Bickl, Andreas; Sedlacek, Lucia; Schwarzkopf, Larissa; Jenny Cisneros Örnberg; Loy, Johanna K	2023	BMC Public Health	#214
**Gambling Harm-Minimisation Tools and Their Impact on Gambling Behaviour: A Review of the Empirical Evidence**	Riley, Ben J; Oakes, Jane; Lawn, Sharon	2024	International Journal of Environmental Research and Public Health	#36
**Gamblers' perceptions of responsibility for gambling harm: a critical qualitative inquiry**	Marko, Sarah; Thomas, Samantha L; Robinson, Kim; Daube, Mike	2022	BMC Public Health	#360
**Responsible product design to mitigate excessive gambling: A scoping review and z-curve analysis of replicability**	McAuliffe, William H B; Edson, Timothy C; Louderback, Eric R; LaRaja, Alexander; LaPlante, Debi A	2021	PLoS One	#406
**Probing the Role of Digital Payment Solutions in Gambling Behavior: Preliminary Results From an Exploratory Focus Group Session With Problem Gamblers**	Lakew, Nathan; Jonsson, Jakob; Lindner, Philip	2024	JMIR Human Factors	#46
**What is known about population level programs designed to address gambling-related harm: rapid review of the evidence**	Clune, Samantha; Ratnaike, Deepika; White, Vanessa; Donaldson, Alex; Randle, Erica; Paul O'Halloran; Lewis, Virginia	2024	Harm Reduction Journal	#52
**Avoiding gambling harm: An evidence-based set of safe gambling practices for consumers**	Hing, Nerilee; Browne, Matthew; Russell, Alex M T; Rockloff, Matthew; Rawat, Vijay; Nicoll, Fiona; Smith, Garry	2019	PLoS One	#613
**The total consumption model applied to gambling: Empirical validity and implications for gambling policy**	Rossow Ingeborg	2019	Nordic Studies on Alcohol and Drugs	#623
**Strategies to customize responsible gambling messages: a review and focus group study**	Gainsbury, Sally M; Abarbanel, Brett L L; Philander, Kahlil S; Butler, Jeffrey V	2018	BMC Public Health	#711
**Understanding the business versus care paradox in gambling venues: a qualitative study of the perspectives from gamblers, venue staff and counsellors**	Riley, Ben J; Orlowski, Simone; Smith, David; Baigent, Michael; Battersby, Malcolm; Lawn, Sharon	2018	Harm Reduction Journal	#719
**A Systematic Review on Intervention Treatment in Pathological Gambling**	Moreira, Diana; Dias, Paulo; Azeredo, Andreia; Rodrigues, Anabela; Leite, Ângela	2024	International Journal of Environmental Research and Public Health	#76
**Public attitudes towards gambling product harm and harm reduction strategies: an online study of 16-88 year olds in Victoria, Australia**	Thomas, Samantha L; Randle, Melanie; Bestman, Amy; Pitt, Hannah; Bowe, Steven J; Cowlishaw, Sean; Daube, Mike	2017	Harm Reduction Journal	#772
**A comparison of two GameSense implementation approaches: How program awareness and engagement relate to gambling beliefs and behaviors**	Louderback, Eric R.; Gray, Heather M.; LaPlante, Debi A.; Abarbanel, Brett; Bernhard, Bo J.	2022	Journal of Gambling Studies	#847
**Assigning responsibility for gambling-related harm: Scrutinizing processes of direct and indirect consumer responsibilization of gamblers in Sweden**	Alexius, Susanna	2017	Addiction Research & Theory	#852
**Association between public opinion of gambling policies, gambling behavior and demographics: A national survey in Finland**	Selin, Jani; Raisamo, Susanna	2021	International Gambling Studies	#854
**Balancing conflicting interests: Stakeholders' interpretations of 'moderation' in swedish gambling advertising legislation**	González Díaz, Katya; Cisneros Örnberg, Jenny; Reitan, Therese	2024	International Gambling Studies	#856
**Bookmakers and a duty of care: Customers' views in England**	Brooks, Graham; Sparrow, Paul	2016	Journal of Gambling Studies	#858

**Consumer protection in licensed online gambling markets in France: The role of responsible gambling tools**	Marionneau, Virve; Järvinen-Tassopoulos, Johanna	2017	Addiction Research & Theory	#860
**Corporate social responsibility in the gambling industry: A systematic review and conceptual framework**	Tetrevova, Libena	2023	International Gambling Studies	#863
**Critiquing the Reno Model I-IV international influence on regulators and governments (2004–2015)—The distorted reality of 'responsible gambling'**	Hancock, Linda; Smith, Garry	2017	International Journal of Mental Health and Addiction	#866
**Expert by experience engagement in gambling reform: Qualitative study of gamblers in the United Kingdom**	Nyemcsok, Christian; Pitt, Hannah; Kremer, Peter; Thomas, Samantha L.	2022	Health Promotion International	#869
**From self-regulation to regulation—An analysis of gambling policy reform in Finland**	Selin, Jani	2016	Addiction Research & Theory	#872
**Global limit setting as a responsible gambling tool: What do players think?**	Auer, Michael; Reiestad, Sigrun Høvik; Griffiths, Mark D.	2020	International Journal of Mental Health and Addiction	#878
**Is ‘‘pop-up’’ messaging in online slot machine gambling effective as a responsible gambling strategy?**	Auer, Michael; Malischnig, Doris; Griffiths, Mark	2014	Journal of Gambling Issues	#882
**Measuring responsible gambling amongst players: Development of the Positive Play Scale**	Wood, Richard T. A.; Wohl, Michael J. A.; Tabri, Nassim; Philander, Kahlil	2017	Frontiers in Psychology	#883
**Reaching out to big losers: A randomized controlled trial of brief motivational contact providing gambling expenditure feedback**	Jonsson, Jakob; Hodgins, David C.; Munck, Ingrid; Carlbring, Per	2019	Psychology of Addictive Behaviors	#889
**Responsible gambling in practice: A case study of views and practices of Swedish oriented gambling companies**	Forsström, David; Örnberg, Jenny Cisneros	2019	Nordic Studies on Alcohol and Drugs	#895
**Sense or sensibility—Ideological dilemmas in gamblers' notions of responsibilities for gambling problems**	Samuelsson, Eva; Örnberg, Jenny Cisneros	2022	Frontiers in Psychiatry	#897
**The effect of loss-limit reminders on gambling behavior: A real-world study of Norwegian gamblers**	Auer, Michael; Hopfgartner, Niklas; Griffiths, Mark D.	2018	Journal of Behavioral Addictions	#899
**The inclusion of health concerns in Swiss gambling legislation: An opportunity to access industry data**	Carlevaro, Tazio; Lischer, Suzanne; Sani, Anna-Maria; Simon, Olivier; Tomei, Alexander	2017	International Gambling Studies	#904
**A measurement scale of corporate social responsibility in gambling industry**	Luo, JM	2018	Journal of quality assurance in hospitality & tourism	#912
**Analyzing Consumer Protection for Gamblers Across Different Online Gambling Operators: A Replication Study**	Catania, M; Griffiths, MD	2023	International journal of mental health and addiction	#914
**An Empirical Study of the Effect of Voluntary Limit-Setting on Gamblers' Loyalty Using Behavioural Tracking Data**	Auer, M; Hopfgartner, N; Griffiths, MD	2021	International journal of mental health and addiction	#917
**An Analysis of Consumer Protection for Gamblers Across Different Online Gambling Operators in Ireland: A Descriptive Study**	Cooney, C; Columb, D; Costa, J; Griffiths, MD; O'Gara, C	2021	International journal of mental health and addiction	#925
**The role of financial institutions in gambling**	Swanton, TB; Gainsbury, SM; Blaszczynski, A	2019	International gambling studies	#934
**Rating the Suitability of Responsible Gambling Features for Specific Game Types: A Resource for Optimizing Responsible Gambling Strategy**	Wood, RTA; Shorter, GW; Griffiths, MD	2014	International journal of mental health and addiction	#962
**Responsible gambling training in Ontario casinos: employee attitudes and experience**	Quilty, LC; Robinson, J; Blaszczynski, A	2015	International gambling studies	#966
**Beyond Reno: a Critical Commentary on Hancock and Smith**	Abbott, MW	2017	International journal of mental health and addiction	#975
**Public health approaches to gambling: a global review of legislative trends.**	Ukhova, Daria; ; Marionneau, Virve; Nikkinen, Janne; Wardle, Heather	2024	The Lancet. Public health	#999

Table 3: Covidence data extraction sheet

Categories	Definition	Category field type
**Manuscript meta information**		
Year	Year of publication	Text field
Author(s)	Manuscript author(s)	Text field
Title	Title of the manuscript	Text field
Research objective	Research question (s) of the study	Text field
Study design	Methodology used/sample description/review type	Text field
**Duty of care/RG measure and findings**		
Themes/Focus	Duty of care and RG approach discussed/evaluated. This iterative process simultaneously optimises the check box lists while also used as categorising the manuscripts. Whenever, the list is changes, the Covidence app reset the categorization process, hence process restarted for all manuscripts.	Check box<br>○ Training and customer service readiness<br>○ Risk Identification and Monitoring<br>○ Players' protection and safeguards<br>○ Limit setting and control<br>○ Proactive engagement with players<br>○ Intervention follow up and escalating<br>○ Game design features<br>○ Access to information and awareness
Implementation/approaches	A summary of how the duty of care or RG measure was implemented within each study. Summaries developed from this examination later used to analysis the Action Plan quality in each identified duty of care themes. The input also was used to create the scoring code criteria.	Text field
Relevancy	Relevancy for the current review or context of duty of care.	Text field
Key concepts and markers	This field presents key concepts and markers associated with each theme that can be used to identify and analyse gambling behavior and take appropriate duty of care actions. It can, for example, include indicators that help distinguish behavioral patterns or risk factors within gambling activities.	Text field

Table 5. Conceptual literature review result – Duty of care

Theme	Description	Sub-theme	Key Concepts and Markers	Example of implementations
**Risk Identification and Monitoring**	Risk identification and monitoring in the gambling context would require analyzing player behavior to detect signs of problem gambling. This includes using data analytics and predictive models to identify high-risk patterns and providing real-time alerts to both players and staff. The goal is to ensure early intervention and maintain a safe gambling environment.	▪ Pre-determined Risk assessment procedures<br>▪ Individual tailored assessment, profiling, and inferring mechanism of gambling risk<br>▪ Behavior tracking systems<br>▪ Risk detection tools<br>▪ Real-time tracking	Behavior/product use Patterns: Frequency, intensity, active days, gambling duration, bet amounts, speed of playing, trajectories in gambling frequency and intensity, and chasing loss.<br>Financial Indicators: payment markers, Deposit amounts and frequency, total wagered, largest losses and associated created variables, bet, withdrawal, account depletion, number of credit cards.<br>Communication and Interaction: request to RG settings, email communication/content, bonus requests.<br>Other anomalies: time of day, day of the week, breadth of product variation use, frequency in risky product selection	Routine screening, tracking transactions, behavior analytics (session, frequency, stake amounts), real-time prediction models, periodic survey assessments, automated risky gambling assessment systems
**Players' protection and safeguards**	Player protection and safeguards in gambling are measures to minimize harm and ensure player well-being. These may include self-exclusion, credit bans, age verification, and ethical advertising to promote responsible gambling. Financial security is prioritized by protecting player funds and limiting or no bonuses to reduce exploitation.	▪ Self-exclusion<br>▪ Credit ban<br>▪ Age verification<br>▪ One-time bonus<br>▪ Active stake/bet amount choice<br>▪ Moderation in advertising gambling products<br>▪ Safeguard Players fund	Gambling pause, self and mandatory exclusions (immediate, limited, until further notice), popup messages, alerts (incl. mode of display, placement, content, messages design, active removal by the gambler), age limits, limited marketing, promoting active bet choices	Harm Reduction plans: Low maximum bets, detection systems with a forced exclusion, no bonus after registration, and withdrawal regulations.<br>RG tools: self-exclusion tools cooling off periods, links to gambling filtering software, bank meter, clock-display, cash display.<br>Alerts and popups: Alters msg, graphic/interactive messages, automated alerts.<br>Feedback and Support Systems: Active feedback loop, customer redress programs, Dedicated RG page
**Limit setting and control**	Limit setting and control involve implementing tools that help players manage their activities to maintain responsible behavior. These tools include setting limits on funds, withdrawals, and spending, as well as features like budget calculators, deposit caps, and pre-	▪ Login session limit<br>▪ Game specific limits<br>▪ Account-based time limit	General gambling duration and session limits, bet and withdrawal limit, loss, limits, budget, precommitments	Systems that enable limits: access to funds, withdrawals, re-bet wins, voluntary limit<br>Safety tools: budget calculator, mandatory limit deposit (day, week, month), pre-commitment

	commitment options. Additional safeguards, such as session limits, game-specific restrictions, and automated alerts, further enhance player protection and promote healthier gambling habits.	▪ Limit automated alerts<br>▪ Bet and withdrawal limits		
**Proactive engagement**	Proactive engagements by gambling operators emphasize encouraging self-assessment and reflection through tools like self-assessment tests and personalized feedback. They provide tailored interactions, such as affordability limits and strategy suggestions, to promote self-control and responsible gambling practices. Operators also deliver educational programs, prevention initiatives, and targeted messages, including RG check-in prompts, warnings, and affordability reminders, personalized for different player groups, such as young adults with insights into play behavior and long-term outcomes.	▪ Encouraging self-assessment and reflection<br>▪ Feedback and active contact based on behavior<br>▪ Education and public message<br>▪ Personalized notifications/warnings	RG check-in prompts, self-control strategy suggestions such as limit suggestion, prevention programs, Affordability limits, tailored interactions (e.g., young adults: report about their play and expertise. 'Skill' game gamblers: Odds of winning and outcomes over time), self-assessment test, educational programs	Personalized engagements: vulnerable population (e.g., age), reminders, education, warning messages, pop-up messages about the time and money spent on gambling, self-reprisal messages<br>Active contacts: via telephone, letter, or email based on gambling behavior.<br>Player Education and Awareness: inform players about the harms of gambling, normative messaging of 'getting to know' of your RG tool, engaging/gamified players education, training gamblers, training courses to gamblers with intervention providers, campaigns and programs about gambling fallacies, animated educational video
**Access to information and Awareness**	Access to information and awareness dimension means making sure that players have easy access to their gambling activities to assess their gambling behavior, encouraging responsible gambling and informed decisions. It requires consistently storing relevant details such as winnings, losses, and log-in duration. Additionally, offering information about responsible gambling (RG) tools, easy access to gambling account history, and providing industry data for research, along with transparency and public reports, further enhance player awareness and support responsible gambling practices.	▪ Storing relevant information at all time<br>▪ Information on winnings<br>▪ Information on losses<br>▪ Log-in duration information	Community empowerment, transparency, encourage accessing gambling habit information, monetary reminder, Gambling diary	Tools and Resources: information about the RG tools offerings, easy accessibility of gambling account history<br>Research and Transparency: Providing industry data for research, transparency and public report

**Follow-up interventions and escalation of severity levels**	Follow-up interventions in gambling involve ongoing communication and personalized contacts to enhance the effectiveness of responsible gambling (RG) measures. Operators follow a structured plan which may include interventions ranging from feedback and tailored recommendations to account suspensions and therapeutic referrals, ensuring a tiered approach to protecting players.	▪ Follow-up communication and contacts<br>▪ Follow-up plan of RG measures<br>▪ Referral to Clinical care	Referrals, gambling problem escalations, account suspension, CBT, measuring positive play	Personalized intervention, referral to treatment services, collaboration with prevention centers, level-based intervention from feedback to therapeutic recommendation
**Product offering selection strategy and transparency**	This dimension describes gambling operators' requirement to clearly communicate the risks associated with game features, the chances of winning, and the rules for each game. Additionally, it requires them to provide educational resources about risky games on their RG pages to help players make informed decisions. Finally, they should implement a standardized product selection strategy and use covert structural tools to steer players toward healthier gambling behaviors.	▪ Mechanics and feature restriction/modification<br>▪ RG game design principles<br>▪ Game selection criteria<br>▪ Game information in RG pages	Transparency, autoplay, clock display, cash display, jackpot expiry, game test, odds, and speed consideration, certified randomness generator, healthy reward system design	*Transparency and Risk Information*: transparency to game feature risk, chances of winning rules for games, information aids<br>Educational Resources: educational resources regarding risky games on RG pages<br>Evaluation and Testing: Independent risk evaluation of games before procurement, product testing protocol in real life (as some users respond differently)<br>Product Strategy: Standardized product selection strategy, covert structural tools to steer toward healthy gambling, In-game notifications
**Training and customer service readiness**	Gambling operators must train customer service staff to recognize and address risky gambling behavior through compulsory RG courses and communication skill lessons. Staff should be equipped with reporting and escalating mechanisms and trained on identifying risky gambling behavior and using RG tools. Additionally, CS units should be provided with RG manuals and education on the duty of care calls and escalation techniques.	▪ Staff training to address/recognize risky behavior<br>▪ Staff training on customer contact and talking points<br>▪ RG skills across customer service staff	RG tool training, RG manuals, CS duty of care calls education, training on escalation techniques	Compulsory unit of RG courses, RG-oriented customer service communication, staff safety, reporting/escalating mechanism/system, identifying risky gambling behavior training programs

Table 6. Scoring code book

Rubric Dimensions	Explanation	Scoring question	0 = Missing	1 = Poor	2 = Fair	3 = Good	4 = Excellent
**Risk identification and Monitoring**	Risk identification and monitoring in the gambling context would require analysing player behavior to detect signs of problem gambling. This includes using data analytics and predictive models to identify high-risk patterns and providing real-time alerts to both players and staff. The goal is to ensure early intervention and maintain a safe gambling environment.	To what extent does the action plan have the provision to effectively identify and monitor risk behaviors, has an established mechanisms to mitigate harm, and utilise data analytics and predictive models for early intervention and a safe gambling environment?	The plan does not address strategies for identifying or monitoring risk behaviors and makes no mention of data analytics, predictive models, real-time alerts, or early intervention.	The plan suggests incoherent strategies for identifying and monitoring risk behaviors. There is minimal use of data analytics or predictive models, and real-time alerts or early intervention are absent.	The plan includes basic methods for risk identification, with some reference to data analytics, but lacks detail or specificity. Monitoring efforts are inconsistent, and early intervention strategies are not well defined. There is very minimal efforts to use predictive models or real-time alerts.	The plan outlines clear and actionable strategies for identifying high-risk behaviors, incorporating data analytics and predictive models. Real-time alerts are set up for both players and staff, and early intervention measures are in place to manage at-risk behaviors.	The plan has a comprehensive and systematic approach to risk identification and monitoring, using advanced data analytics, predictive models, and real-time alerts for early intervention. It includes a well-defined, proactive system for player engagement and ongoing monitoring to ensure a safe gambling environment.
**Players' protection and safety procedures**	Player protection and safeguards in gambling are measures to minimize harm and ensure player well-being. These may include self-exclusion, credit bans, age verification, and ethical advertising to promote responsible gambling. Financial security is prioritized by protecting player funds and limiting bonuses to reduce exploitation.	To what extent does the proposed action plan implement effective player protection and safeguards to minimize harm, including measures such as self-exclusion, credit bans, age verification, ethical advertising, and financial security to ensure player well-being?	The plan does not address players protection and safeguards measures, and harm minimalization measures are missing.	The plan lacks clearly established strategy for player protection and safeguards, with little provision for self-exclusion, credit bans, or age verification. Ethical advertising practices, dedicated RG pages, automated alerts, and financial security measures are briefly discussed.	The plan includes basic player protection measures, such as self-exclusion and age verification, but they are limited in scope or lack detailed implementation. Financial security measures, such as protecting player funds and limiting bonuses, are minimally addressed, with some gaps in ethical advertising practices and RG page description.	The plan outlines well-defined measures for player protection, including self-exclusion, credit bans, age verification, and ethical advertising. Financial safeguards, such as fund protection and bonus limitations, are effectively integrated with a well-developed dedicated RG page.	The plan offers a comprehensive player protection, featuring robust self-exclusion options, credit bans, cool off periods, strict age verification, and well-developed RG pages. Financial security measures are thoroughly implemented, ensuring the protection of player funds and minimizing risks through clear and ethical safeguards.

**Limit setting and control**	Limit setting and control involve implementing tools that help players manage their activities to maintain responsible behavior. These tools include setting limits on funds, withdrawals, and spending, as well as features like budget calculators, deposit caps, and pre-commitment options. Additional safeguards, such as session limits, game-specific restrictions, and automated alerts, further enhance player protection and promote healthier gambling habits.	To what extent does the proposed action plan enable to deliver a comprehensive and effective limit-setting and control measures to help players manage their gambling time and money spent behavior?	The plan does not address limit-setting and control measures or strategies for managing gambling duration and spending behavior.	The plan lacks strategic planning for limit-setting and control approach. Minimal mechanisms are outlined for session limits, budget tools, or voluntary limits, and there is no integration of safety tools such as automated alerts or mandatory deposit limits.	The plan delivers basic limit-setting measures, such as general gambling session limits and voluntary budget tools, but they lack comprehensive features. Safety tools, such as automated alerts or mandatory deposit limits, are not fully integrated.	The plan has a well-defined and actionable strategies for limit-setting, including general and game-specific session limits, loss limits, pre-commitment options, and re-betting wins restrictions. Unique safety tools, such as budget calculators, and automated alerts, are included as a provision.	The plan shows a comprehensive framework for limit-setting and control, incorporating detailed measures such as gambling session, game-specific, and withdrawal limits, along with robust tools like budget calculators, mandatory deposit limits, and automated alerts. There is a strategy on how all features are integrated to ensure healthy time and money spending habits.
**Proactive engagement**	Proactive engagements by gambling operators emphasize encouraging self-assessment and reflection through tools like self-assessment tests and personalized feedback. They provide tailored interactions, such as affordability limits and strategy suggestions, to promote self-control and responsible gambling practices. Operators also deliver educational programs, prevention initiatives, and targeted messages, including RG check-in prompts, warnings, and affordability reminders, personalized for different player groups, such as young adults with insights into play behavior and long-term outcomes.	To what extent does the proposed action plan incorporate proactive strategies to encourage self-assessment, promote responsible gambling, and provide tailored support to diverse player groups?	The plan does not include any proactive engagement strategies, tools for self-assessment, personalized feedback, tailored interactions, educational programs, prevention initiatives, or targeted messages.	The plan lacks proactive engagement strategies, with very minimal tools for self-assessment, personalized feedback, or tailored interactions. Educational programs, prevention initiatives, and targeted messages are insufficiently addressed.	The plan includes some proactive engagement measures, such as basic self-assessment tools and general educational programs, but limited planning is sought to affordability reminders, strategy suggestions, or tailored interventions for specific player groups.	The plan has clear and actionable proactive engagement strategies, including self-assessment, personalized feedback, and targeted RG check-in prompts. Educational programs and prevention initiatives are effectively designed, with some level of tailoring for different player groups.	The plan provides a comprehensive and innovative approach to proactive engagement, incorporating tools such as self-assessment, personalized feedback, affordability reminders, and strategy suggestions. It has a well-designed RG promotion program, prevention campaigns, and tailored interactions for diverse player groups.

**Access to information and Awareness**	Access to information and awareness dimension means making sure that players have an easy access to their gambling activities to assess their gambling behaviour, encouraging responsible gambling and informed decisions. It requires consistently storing relevant details such as winnings, losses, and log-in duration. Additionally, offering information about responsible gambling (RG) tools, easy access to gambling account history, and providing industry data for research, along with transparency and public reports, further enhance player awareness and support responsible gambling practices.	To what extent does the proposed action plan ensure access to information, effective data storage, and awareness to support responsible gambling through transparency, accessible tools, and community empowerment?	The plan does not address storing or providing access to information about RG tools, gambling account history, or player activity data, and makes no mention of transparency, public reporting, or tools for community empowerment.	"The plan briefly mentions provisions for storing or accessing RG tools, gambling account history, and player activity data, but lacks clear strategy. Transparency, public reporting, and community empowerment tools are minimally addressed."	The plan includes basic measures for storing and providing information, such as limited accessibility to gambling account history and RG tools. Some aspects of transparency, such as public reporting, are present but lack detail, and efforts to promote community empowerment or habit-tracking tools like gambling diaries are minimal.	The plan outlines effective storage and access to relevant information, including gambling account history and player activity (e.g., winnings, losses, and log-in duration). It incorporates transparency through public reporting, promotes community empowerment, and encourages self-awareness with tools such as gambling diaries. Some provisions are included to share industry data for research.	The plan offers a comprehensive strategy for storing and enabling access to gambling-related information, ensuring easy direction to RG tools, account history, and player activity data. It has a strategy for promoting transparency through public reporting, empowers the community, and encourages responsible gambling with direct access to habit-tracking tools. Additionally, it supports industry collaboration by providing data for research.
**Follow-up Interventions and escalation of problem gambling severity Levels**	Follow-up interventions in gambling involve ongoing communication and personalized contacts to enhance the effectiveness of responsible gambling (RG) measures. Operators follow a structured plan which may include interventions ranging from ongoing feedback and tailored recommendations to account suspensions and therapeutic referrals, ensuring a tiered approach to protecting players.	To what extent does the proposed action plan provide a structured system of follow-up interventions and escalation processes to address problem gambling effectively and protect players?	The plan does not address follow-up interventions, escalation processes, personalized contacts, or tiered approaches to address problem gambling severity.	The plan lacks a clear structure for follow-up interventions and escalation processes. There is minimal planning for ongoing communication, personalized contacts, or tiered approaches to address problem gambling severity.	The plan includes basic follow-up measures, such as general feedback or occasional communication, but lacks detailed intervention strategies or a structured system for escalating problem gambling cases. Personalized contacts and tailored recommendations are limited.	The plan outlines a structured follow-up system with ongoing communication and personalized contacts. It includes tiered interventions such as tailored recommendations, feedback, account suspensions, and therapeutic referrals.	The plan provides a comprehensive and well-defined system for follow-up interventions, combining regular communication, personalized contacts, and detailed feedback. It includes a robust, tiered escalation process with clear pathways for tailored recommendations, account suspensions, and therapeutic as well as support group referrals.

**Product offering selection strategy and transparency**	This dimension describes gambling operators' requirement to clearly communicating the risks associated with game features, the chances of winning, and the rules for each game. Additionally, it requires them to provide educational resources about risky games on their RG pages to help players make informed decisions. Finally, they should implement a standardized product selection strategy and use covert structural tools to steer players toward healthier gambling behaviors.	To what extent does the proposed action plan ensure a standardized game offering selection strategy and communicate game risks effectively to promote healthier gambling behaviors?	The plan does not address nor provide any strategy in the context of product and game features related issues, including communication of risk.	"The plan briefly mentions risks associated with specific games but lacks a clear communication strategy of risks, chances of winning, or rules for the offered games. It also lacks a standardized product selection strategy to encourage healthier gambling behaviors."	The plan includes basic communication about game rules and chances of winning but provides limited information about risks associated with some game features. Educational resources about risky games are minimal, and the product selection strategy is inconsistent or insufficiently defined.	The plan communicates game risks, winning chances, and rules clearly and provides accessible educational resources about risky games on RG pages. It includes a standardized product selection strategy and employs some covert structural tools to steer players toward healthier gambling behaviors.	The plan demonstrates a comprehensive and transparent approach to product offering and selection. It clearly communicates game risks, chances of winning, and supported by robust educational resources about risky games. A well-defined product selection strategy is in place and including the use covert structural tools to promote informed decisions and encourage safer gambling behaviors.
**Training and customer service readiness**	Gambling operators must train customer service staff to recognize and address risky gambling behavior through compulsory RG courses and communication skill lessons. Staff should be equipped with reporting and escalating mechanisms and trained on identifying risky gambling behavior and using RG tools. Additionally, CS units should be provided with RG manuals and education on the duty of care calls and escalation techniques.	To what extent does the proposed action plan ensure comprehensive training and readiness of customer service staff to recognize, address, and escalate risky gambling behaviors effectively?	The plan does not address customer service training on recognizing risky gambling behavior or using RG tools. There are no provisions for reporting, escalation mechanisms, or educational resources for customer service staff.	The plan briefly mentions customer service training but lacks compulsory RG courses or communication skill lessons. It does not sufficiently address reporting, escalation mechanisms, or the provision of RG manuals or education on duty of care calls.	The plan includes basic training strategy for customer service staff, such as general awareness of RG tools and identification of risky gambling behaviors but lacks structured training. RG manuals, education on duty of care calls, reporting mechanisms, and comprehensive escalation protocols are limited	The plan incorporates well-structured training for customer service staff, covering recognition of risky gambling behaviors, effective use of RG tools, and escalation procedures. Staff are provided with RG manuals and trained in duty of care calls and communication skills.	The plan provides a robust and detailed framework for continuous training customer service staff, including compulsory RG courses, advanced communication skills, and comprehensive escalation protocols. Staff are fully equipped with RG manuals, reporting mechanisms, and education on duty of care calls.

Table 7. Rubrics Dimension Results

Action Plans	Risk identification and monitoring	Players' protection and safety procedures	Limit setting and control	Proactive engagement	Access to information and Awareness	Follow-up Interventions and escalation of problem gambling severity Levels	Product offering selection strategy and transparency	Training and customer service readiness	Score (0 – 4)	Alignment percentage
AP_01	3	2	2	1	1	3	1	3	2	50%
AP_02	3	3	3	1	2	2	1	4	2.37	59%
AP_03	1	1	2	0	1	1	0	2	1	25%
AP_04	4	4	4	3	3	3	0	4	3.12	78%
AP_05	2	1	2	1	1	2	1	2	1.5	38%
AP_06	2	2	2	2	1	1	0	0	1.25	31%
AP_07	4	4	3	3	3	3	0	4	3	75%
AP_08	4	4	3	3	3	2	4	3	3.25	81%
AP_09	1	1	2	1	1	0	0	1	0.87	22%
AP_10	4	4	4	3	3	4	0	2	3	75%
AP_11	4	3	2	2	2	2	1	3	2.37	59%
AP_12	2	2	1	0	0	0	1	0	0.75	19%
AP_13	4	2	3	2	2	2	0	2	2.12	53%
AP_14	2	2	2	1	0	0	0	1	1	25%
AP_15	1	1	2	0	0	0	0	0	0.5	13%
AP_16	1	1	0	0	0	2	0	0	0.5	13%
AP_17	3	3	2	2	0	2	0	3	1.87	47%
AP_18	2	2	1	1	0	3	0	0	1.12	28%
AP_19	4	4	3	2	2	4	0	4	2.87	72%
AP_20	4	4	3	3	2	3	0	3	2.75	69%
AP_21	0	2	0	0	0	0	0	0	0.25	6%
AP_22	4	4	3	3	4	3	0	0	2.62	66%
AP_23	1	1	1	0	0	1	0	0	0.5	13%
AP_24	1	2	2	1	1	2	1	1	1.37	34%
AP_25	4	4	4	3	4	3	0	4	3.25	81%
AP_26	4	3	4	2	4	3	0	4	3	75%

AP_27	3	4	4	3	3	3	3	3	3.25	81%
AP_28	3	3	3	1	2	1	0	2	1.87	47%
AP_29	1	1	1	1	0	1	0	0	0.62	16%
AP_30	4	4	4	4	4	4	2	3	3.62	91%
AP_31	3	3	3	2	1	2	0	2	2	50%
AP_32	4	4	4	3	3	4	2	4	3.5	88%
AP_33	1	1	2	0	1	1	0	0	0.75	19%
AP_34	3	3	4	2	1	2	0	1	2	50%
AP_35	1	0	1	1	0	2	0	0	0.62	16%
AP_36	2	2	2	1	0	0	1	2	1.25	31%
AP_37	2	3	3	2	1	3	0	3	2.12	53%
AP_38	3	2	3	2	2	1	0	2	1.87	47%
AP_39	3	3	2	2	2	3	0	3	2.25	56%
AP_40	4	4	4	3	2	4	2	4	3.37	84%
AP_41	1	4	3	1	1	0	1	1	1.5	38%
AP_42	4	3	3	3	2	3	0	4	2.75	69%
AP_43	4	4	3	2	2	3	0	4	2.75	69%
AP_44	3	3	3	2	3	3	0	2	2.37	59%
AP_45	2	2	3	0	1	2	0	3	1.62	41%
AP_46	2	4	2	2	1	2	0	2	1.87	47%
AP_47	2	2	2	1	0	2	2	3	1.75	44%
AP_48	4	2	3	0	1	2	0	1	1.62	41%
AP_49	4	4	4	2	2	4	0	4	3	75%
AP_50	2	2	2	1	1	1	0	0	1.12	28%
AP_51	2	2	3	1	2	1	0	0	1.37	34%
AP_52	3	3	2	2	2	3	0	2	2.12	53%
